# Disparities in COVID-19 Information Sources and Knowledge in South Korea

**DOI:** 10.3390/ijerph19095198

**Published:** 2022-04-25

**Authors:** Sou Hyun Jang

**Affiliations:** Department of Sociology, Korea University, 145 Anam-ro, Anam-dong, Seongbuk-gu, Seoul 02841, Korea; soujang@korea.ac.kr

**Keywords:** COVID-19, pandemic, South Korea, sense-making theory, information-seeking behavior, information source

## Abstract

Applying Dervin’s sense-making theory, this study aims to examine the factors associated with the numbers and types of COVID-19 information sources, and the association between information sources and knowledge during the COVID-19 pandemic in South Korea. An online survey was conducted among adults (19–69 years old) in December 2020. Ordinary least squares (OLS) and logistic regression were conducted to examine (1) the associated factors with the numbers and types of COVID-19 information sources, and (2) whether the number and types of COVID-19 information sources predict the correct COVID-19 knowledge. On average, the participants utilized five different sources to find COVID-19 information. The information need was related to the number and type of information sources, while the information barrier was only related to the number of sources. Participants who utilized more sources and who utilized online sources were more likely to possess the correct knowledge regarding COVID-19 while utilizing the government website; however, doctors, as a source, were negatively related to COVID-19 knowledge. There should be more support for individuals with lower socioeconomic status, as they tend to look for fewer sources, while finding more sources is positively related to better COVID-19 knowledge.

## 1. Introduction

As the coronavirus disease 2019 (COVID-19), which was first identified in December 2019 and announced as a pandemic by the World Health Organization (WHO) in 2020, is novel, information about the virus had to be easily accessible. Most studies have found that individuals have utilized various sources for information on COVID-19. However, traditional media and social media (for example, Twitter, Facebook, YouTube) have been more widely used as sources of COVID-19 information than interpersonal sources, while disparities exist among people of diverse sociodemographic characteristics [1,2,3,4,5]. For example, female, younger, and those with a high school or lower education were more likely to search for COVID-19 information via online media than their counterparts who were male, older, and those with a college or higher degree in the U.S. [1]. Understanding the sources of COVID-19 information could prove imperative because it is known to be associated with COVID-19 knowledge [1].

South Korea (hereinafter Korea) is a country that has been praised for flattening the curve in the early COVID-19 outbreak in 2020 [6]. However, to the best of my knowledge, no study has examined the sources of COVID-19 information seeking in Korea. Instead of focusing on COVID-19 information-seeking and its relationship to correct COVID-19 knowledge, research on Korea during the COVID-19 pandemic has primarily focused on factors related to preventive measures, finding a positive relationship between preventive measures and COVID-19 knowledge [7], as has been found in other countries such as the Philippines [8] and Pakistan [9].

To understand the information-seeking behavior, Dervin [10], in his sense-making theory, indicates that a “situation” in which an individual has a knowledge “gap” could lead them to seek “help”. While the sense-making theory has been widely applied in the field of information-seeking behavior [11], scholars later suggested an expanded model with other components to understand information-seeking, such as “need” and “barrier”, to be considered [12]. COVID-19 is a new “situation” that forces all individuals to uncover relevant information to gain the knowledge they have not encountered before.

Several studies have applied the sense-making theory to investigate an individual’s information-seeking behavior during the COVID-19 pandemic. Most people, particularly the vulnerable (for example, older persons in remote regions), experience unmet “needs” and “barriers” to accessing information during the COVID-19 pandemic [13]. In addition, young individuals are concerned for their vulnerable elderly parents or relatives [14]. According to a study comparing information-seeking among older adults in the U.S. and India, the national context may be important in information-seeking during the COVID-19 pandemic [13]. This study discovered differences in information needs and utilization of information sources between these two groups, implying that the national context may be important in information-seeking during the COVID-19 pandemic. However, how each individual bridges the “gap” by utilizing different numbers and types of information sources has been understudied in the Korean context. Furthermore, the discrepancies in the “outcome” of information-seeking that could result from bridging the gap are less well-known. 

**Figure 1:**
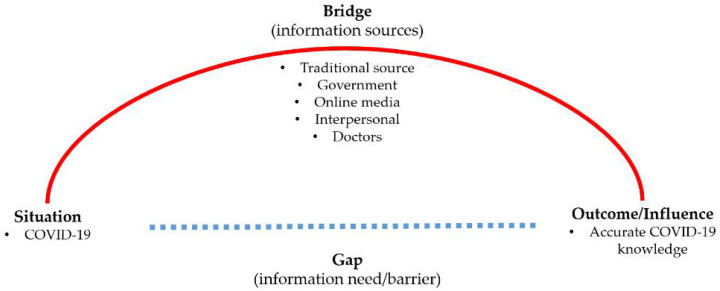
Conceptual Framework of the Study.

Applying Dervin’s sense-making theory (Figure 1), this study examines the relationship between COVID-19 information-seeking and correct COVID-19 knowledge in Korea during the pandemic. It has two main objectives. First, it examines the factors associated with the disparities (regarding the number and type of sources) in COVID-19 information sources by focusing on the information needs and barriers. Finally, it explores whether information-seeking is related to better COVID-19 knowledge during the pandemic. The findings of this study will contribute to the broader literature on information-seeking and policy implications to provide necessary information to those who have incorrect COVID-19 knowledge or are marginalized in acquiring it.

## 2. Materials and Methods

This study analyzed a web survey of 1500 adults 19–69 years of age. The survey was run through Research & Research, a professional survey company in Korea. Quota sampling was used based on age, sex, and area of residence. The survey was conducted in December 2020, during the COVID-19 pandemic. 

The main dependent variable was COVID-19 knowledge, which was measured based on nine statements. The survey respondents were requested to choose an answer among “true”, “false”, and “do not know” each statement (Table 1). Then, these items were converted into either 0 if the answers were incorrect or “do not know” or 1 if the answers were correct. The mean of COVID-19 correct knowledge was 0.558, with a standard deviation of 0.219.

The independent variable is the source of COVID-19 information, especially the number and source types. The survey participants were asked to choose any source of COVID-19 information, while duplicate answers were possible among these 10 sources: TV, radio, newspaper (paper printed), internet news, the Korea Centers for Disease Control and Prevention (KCDC) website, social media, YouTube, family members, friends, and doctors. For the analyses, these 10 sources were sorted into 5 categories: (1) traditional media, such as TV, radio, and paper newspapers; (2) government, and the KCDC website; (3) online media, including internet news, social media, and YouTube; (4) interpersonal sources, comprising family members and friends; (5) doctors. The source has been coded if at least one of the corresponding items was used. The number of information sources was obtained by adding the number of each source (range: 0–10). 

Additionally, information needs and barriers were included as independent variables to predict the number and types of sources of COVID-19 information. To measure information needs, the participants were asked to answer the following question: “How important is the following information to you regarding COVID-19?” The answer options were: “how to” (1) protect myself and my family from the infection; (2) increase immunity; (3) exercise; (4) effectively explain COVID-19 to my family; (5) avoid worries and fears of COVID-19, (6) respond to the COVID-19 infection; (7) know symptoms of COVID-19; and (8) cure COVID-19. Each response was rated on a 5-point Likert scale ranging from “not at all” (1 point) to “very important” (5 points). The average of the points obtained from the eight responses was used, and the Cronbach’s alpha (α) was 0.8829.

The question, “How difficult was the following when you were looking for information related to COVID-19?” was used to measure information barriers in the following 6 aspects: (1) to check whether it is true or fake news, (2) too much information, (3) the absence of reliable information sources, (4) difficulty in understanding due to scientific terms or English, (5) lack of time to find information, and (6) finding the information itself is mentally stressful. Each response was rated on a 5-point Likert scale ranging from “not difficult at all” (1 point) to “very difficult” (5 points). The average of the points obtained from the eight responses was used, and the Cronbach’s alpha (α) was 0.8540.

Control variables included participants’ age, gender (male vs. female), marital status and having kids (unmarried; married without kids; married with kids), educational level (high school or below; college graduates; graduate school), employment type (unemployed; regular worker; temporary worker; self-employed or unpaid family worker), and monthly household income.

The OLS regression and logistic regression were conducted. All analyses were conducted using Stata 17.0 software. The significance level was set at *p* < 0.05.

## 3. Results

### 3.1. Characteristics of Survey Participants

Table 2 presents the characteristics of the survey participants by the source of COVID-19 information-seeking. Considering all participants, the mean age was 44.49 years old with a standard deviation (SD) of 12.96. Approximately half of the participants were female (49.33%), and slightly more were married (58.67%). Most graduated college or had graduate-level education. Slightly less than a quarter were unemployed, while about 55.87% were regular workers, 7.33% were temporary workers, and 9.47% were self-employed and unpaid family workers. Less than one-third of the participants (62.0%) had less than a $5000 monthly household income. Table 2 also shows that the source of COVID-19 information differed according to participants’ sociodemographic characteristics. For example, older participants and those who were married with children relied more on doctors than other sources to seek COVID-19 information than their younger and unmarried counterparts. Female participants reported a higher rate of using the government as the source of COVID-19 information (53.44%) than their male counterparts (46.56%).

### 3.2. Sources of COVID-19 Information

Except for 35 participants (2.3%), the majority utilized more than 1 source to acquire COVID-19 information. On average, the participants used 5 sources (mean = 5.37; SD = 2.15) to obtain COVID-19 information. While multiple sources were available to choose from, most participants reported using TV (92.3%) and internet news (89.0%) as sources of COVID-19 information. Additionally, family members (77.9%) and friends (72.9%) were frequently used as sources. Less than half of the participants used YouTube (44.2%), radio (40.6%), social media (37.8%), KCDC websites (32.9%), and newspapers (27.2%). Doctors (23.0%) were the least popular sources of COVID-19 information.

### 3.3. Factors Associated with the Number and Type of Sources of COVID-19 Information

Table 3 shows factors associated with the number of sources for COVID-19 information-seeking. Both needs for COVID-19 information (Model 1) and barriers to COVID-19 information (Model 2) were positively and significantly related to the number of sources to find COVID-19 information. Specifically, individuals who need more COVID-19 information, and who experience more barriers tended to search for various sources. When standardized coefficients, as well as adjusted R^2^, were compared, information needs were more important than information barriers in predicting the number of sources to search for COVID-19 information (Model 3). The significant relationships between information needs and the number of sources and between information barriers and the number of sources remained after controlling for covariates (Model 4).

In addition to the needs and barriers, survey participants who were married—both with or without kids—were more likely to utilize more sources to seek COVID-19 information than those who were unmarried. Additionally, those of a higher socioeconomic status tended to use more sources; participants with a graduate school degree or higher, who were regular workers, and who have higher household income were more likely to utilize more sources than those with a high school education or below, were unemployed, and had a lower household income.

Table 4 shows that individuals who have higher information needs were more likely to seek COVID-19 information from all sources, except for doctors. They reported higher tendencies to use traditional media, government, online media, and interpersonal sources. In contrast to information needs, the information barrier was only related to seeking COVID-19 information from doctors. Based on the R^2^, the model predicted the utilization of traditional media the best, followed by the online media, interpersonal sources, doctors, and the government as the sources of COVID-19 information.

Aside from information needs, there were differences in the types of participants’ utilization of the source of COVID-19 information based on their demographic characteristics. Women were significantly more likely than men to use government and interpersonal sources. Being older was negatively related to the utilization of the government website to seek COVID-19 information, but positively associated with using doctors as sources of COVID-19. Married participants —regardless of whether they had children—reported higher tendencies to use traditional and interpersonal sources than unmarried ones. Educational attainment was not associated with sources of COVID-19 information, except that participants with a college degree were more likely to use online media than those with a high school education or below. Regular workers tended to use online media) and interpersonal sources more than unemployed participants. Those with a higher income were more likely to use the interpersonal source and doctors than those in the lowest income category.

### 3.4. COVID-19 Information Sources and Correct Knowledge about COVID-19

The relationship between COVID-19 information sources and knowledge of COVID-19 considerably varied according to the knowledge question (Table 5). On the one hand, the number of COVID-19 information sources was positively related to only one type of knowledge: eating kimchi prevented COVID-19. On the other hand, the types of COVID-19 information sources were significantly associated with accurate knowledge about COVID-19, except for one statement (eating or contact with wild animals would result in infection with the COVID-19 virus). The participants who used online media were more likely to answer correctly regarding six different statements (for example, the main clinical symptoms of COVID-19 are fever, coughing, and loss of taste; Currently, there is no cure for COVID-19; COVID-19 virus is airborne; COVID-19 is transmitted through respiratory droplets; COVID-19 spreads from human to human; Not all people with COVID-19 will develop severe symptoms). While participants who used government websites were more likely to have correct knowledge about one statement (distinct from the common cold, nasal congestion, runny nose, and sneezing were less common in persons infected with COVID-19), they were less likely to have accurate knowledge about another statement (COVID-19 spreads from human to human). Participants utilizing doctors as sources of COVID-19 information, however, were less likely to report correct knowledge about three statements (the main clinical symptoms of COVID-19 are fever, coughing, and loss of taste; currently, there is no cure for COVID-19; not all people with COVID-19 will develop severe symptoms.

According to the Pseudo R^2^, the utilization of more and different sources of COVID-19 information predicted the accurate knowledge about the transmission (COVID-19 is transmitted through respiratory droplets; COVID-19 spreads from human to human) and symptoms (the main clinical symptoms of COVID-19 are fever, coughing, and loss of taste; not all people with COVID-19 will develop severe symptoms). 

## 4. Discussion

The current study examined the factors associated with the number and types of COVID-19 information sources, as well as whether the use of information sources is related to correct COVID-19 knowledge, using the sense-making theory, in which individuals seek to bridge knowledge gaps in their knowledge in a specific situation. Overall, the sense-making theory during the COVID-19 pandemic in Korea was applicable; more COVID-19 information needs and barriers were related to the higher number of information sources, and information needs were related to the utilization of different types of information sources. Furthermore, confirming the sense-making theory, an individual searcher who utilized more and various information sources reported higher tendencies to have accurate knowledge about COVID-19.

On average, the survey participants utilized five sources. Additionally, this study found that individuals with more information gaps (such as needs and barriers) tend to utilize more information sources. These findings suggest that each information source might lack “completeness”, in that a single source does not provide enough information about COVID-19; thus, individuals might need to seek additional information from other sources. This implies that providing complete information about COVID-19 is important, as the information provided by different sources might be contradictory.

There were disparities in the numbers seeking COVID-19 related information. Individuals who were married, had a higher education, engaged in regular work, and had higher income were more likely to utilize more sources than those who were unmarried, less educated, unemployed, and had lower income. A higher number of COVID-19 information sources were positively related to the correct COVID-19 knowledge. These findings, in line with previous studies [13,14], imply that more support is needed to provide complete COVID-19 information for these socioeconomically vulnerable populations. Effective health communication campaign (for example, a poster on the wall) aimed these vulnerable populations in various settings, such as workplaces and low-income neighborhood, could be beneficial.

There were also disparities in the types of COVID-19 information sources. Overall, most participants relied on online and traditional media more than other sources, thus confirming the findings of earlier studies [1,15]. Given the research concerning misinformation about COVID-19 dissemination [16,17], especially via online media [15,18,19,20], there should be valid fact-checking regarding the information in online and traditional media to ensure that these usual sources could provide more accurate information. Additionally, as the importance of governments increased during the pandemic [21], more support is needed to promote the utilization of the government website. Furthermore, this study found that having a doctor as a source of COVID-19 information was negatively related to correct COVID-19 knowledge. COVID-19 is an unprecedented event for all, including healthcare professionals. As found earlier [19], COVID-19 misinformation is also prevalent among doctors; more than two-thirds of healthcare professionals, including doctors and practitioners, encounter misinformation about COVID-19. Providing accurate information to healthcare professionals through continuing education programs or top-down information from the KCDC could contribute to disseminating accurate COVID-19 information to the nation.

This study has several limitations that should be addressed in future studies. First, due to data limitations, it was not feasible to determine the kind of information that the participants were seeking. Studies have pointed out that individuals seek not only medical but also non-medical information owing to the detrimental impact of COVID-19 [22]. Participants who want medical information might utilize the KCDC website or doctors more than those who wanted to search for non-medical information. Second, it was not feasible to verify whether the information provided by each source was correct or not. As researchers have pointed out [15,16,18,19,20], misinformation about COVID-19 has been widespread and disseminated, especially via online media. Thus, future studies should examine which source is more likely to convey misinformation and how to combat it. Third, according to the sense-making theory, people’s motivation to find information, communication exchanges, shared meanings, and activities may all play a role in their attempts to bridge knowledge gaps. These factors could not be included owing to data restrictions. Finally, COVID-19-related information that was previously believed to be correct at the onset of the pandemic has since been updated. For example, during the early stages of the pandemic in Korea [23], when the survey for the current study was collected, there was an early consensus that the coronavirus was transmitted by droplets. However, new data suggest that the coronavirus is spread through the air [24]. More investigation on the most recent COVID-19 data is required.

## 5. Conclusions

The current study found that on average, Koreans use an average of five distinct types of information source to find information about COVID-19, with those who face information barriers using a variety of sources. More assistance should be provided to more vulnerable individuals in lower socioeconomic positions, as they tend to seek out fewer sources, despite the fact that having more sources is linked to greater COVID-19 knowledge. Furthermore, because using the government website and doctors to find COVID-19 information was found to be negatively related to correct COVID-19 knowledge, more support for clear communication between the government and citizens, as well as continuing education for doctors about COVID-19, is required. Future studies could examine whether the numbers and types of COVID-19 information are related to COVID-19 preventive behaviors, including COVID-19 vaccination, in addition to COVID-19 knowledge.

## Figures and Tables

**Table 1:** Responses to knowledge item (%).

Knowledge Items	True	False	Do Not Know
1. The main clinical symptoms of COVID-19 are fever, coughing, and loss of post-taste.	**79.07**	6.33	14.60
2. Unlike the common cold, nasal congestion, a runny nose, and sneezing are less common in people infected with COVID-19.	**24.73**	29.20	46.07
3. Currently, there is no cure for COVID-19.	**59.27**	19.47	21.27
4. Eating or contact with wild animals would result in infection with the COVID-19 virus.	38.47	**28.40**	33.13
5. Eating kimchi prevents COVID-19.	10.73	**56.93**	32.33
6. The COVID-19 virus is airborne.	53.87	**27.67**	18.47
7. COVID-19 is transmitted through respiratory droplets.	**85.60**	6.13	8.27
8. COVID-19 spreads from human to human.	**81.73**	9.07	9.20
9. Not all people with COVID-19 will develop severe symptoms.	**68.93**	14.60	16.47

Note: Bold means the correct knowledge.

**Table 2:** Characteristics of 1500 participants with data on COVID-19 sources of information, December 2020, %.

	Source of Information Used					
	Total (*n* = 1500)	Traditional Media (*n* = 1421)	Government (*n* = 494)	Online Media (*n* = 1411)	Interpersonal (*n* = 1269)	Doctor (*n* = 345)
**Age** (mean, SD)	44.49 (12.96)	44.89 (12.84)	44.00 (12.78)	44.61 (12.85)	44.84 (12.85)	48.44 (12.88)
**Sex**						
Male	50.67	49.82	46.56	50.67	48.86	53.04
Female	49.33	50.18	53.44	49.33	51.14	46.96
**Marital status and children**						
Unmarried	41.33	39.62	39.27	41.25	37.83	27.54
Married without children	24.87	25.69	23.28	24.88	26.79	33.33
Married with children	33.80	34.69	37.45	33.88	35.38	39.13
**Educational attainment**						
High school graduates or less	18.27	18.51	18.02	17.51	17.57	19.13
College graduates	72.87	72.77	71.66	73.42	73.44	69.57
Graduate school	8.87	8.73	10.32	9.07	8.98	11.30
**Employment type**						
Unemployed	27.33	26.95	26.11	26.36	26.40	24.93
Regular workers	55.87	56.30)	57.89	56.91	57.60	58.55
Temporary workers	7.33	7.32	6.68	7.37	7.25	5.22
Self-employed and unpaid family workers	9.47	9.43	9.31	9.36	8.75	11.30
**Income**						
Less than $3000	27.13	26.32	24.70	26.22	24.51	20.58
$3000 to less than $5000	34.87	34.98	34.21	35.01	35.46	34.20
$5000 to less than $7000	20.40	20.83	22.47	20.69	20.96	24.06
$7000 or above	17.60	17.87	18.62	18.07	19.07	21.16

SD = Standard Deviation. Note: ₩1 (Korean won) was calculated at approximately $1 for household income.

**Table 3:** Factors associated with the number of sources for seeking COVID-19 information (*n* = 1500).

	Model 1	Model 2	Model 3	Model 4
**Information Needs**	0.25 ***		0.24 ***	0.26 ***
**Information Barriers**		0.10 ***	0.05 *	0.05 *
**Age**				0.03
**Gender** (ref: male)				
Female				0.01
**Marital status and children (ref: unmarried)**				
Married without kids				0.10 **
Married with kids				0.12 ***
**Educational attainment** **(ref: high school or below)**				
College graduates				0.05
Graduate school				0.08 **
**Employment status** **(ref: unemployed)**				
Regular worker				0.11 ***
Temporary worker				0.04
Self-employed or unpaid family worker				0.05
**Income**				0.11 ***
F-value (Degree of Freedom)	103.67 (1,1498)	15.62 (1,1498)	54.25 (2,1497)	18.32 (12,1487)
*p* value	0.000	0.0001	0.000	0.000
Adjusted R^2^	0.0641	0.0097	0.0663	0.1218

Standardized coefficients (beta). *** *p* < 0.001; ** *p* < 0.01; * *p* < 0.05.

**Table 4:** Adjusted odds ratios of factors associated with COVID-19 information source.

	Source of Information Used				
	Traditional Media (*n* = 1421)	Government (*n* = 494)	Online Media (*n* = 1411)	Interpersonal Sources (*n* = 1269)	Doctor (*n* = 345)
**Information Needs**	2.76 ***	1.38 ***	3.26 *	1.64 ***	1.04
**Information Barriers**	0.95	0.98	0.95	1.05	1.20 *
**Age**	1.01	0.98 *	1.01	0.99	1.02 ***
**Sex** (ref: male)					
Female	1.61	1.27 *	1.02	1.60 **	0.33
**Marital status and children (ref: unmarried)**					
Married without children	2.49 *	1.05	0.53	2.98 ***	1.45
Married with children	2.26 *	1.25	0.54 *	1.87 ***	1.40 *
**Educational attainment (ref: high school graduates or below)**					
College graduates	0.78	0.91	1.81 *	1.17	0.90
Graduate school	0.48	1.19	2.21	1.09	1.13
**Employment type (ref: unemployed)**					
Regular workers	1.59	1.15	2.16 **	1.52 *	1.14
Temporary workers	1.38	0.98	1.92	1.35	0.78
Self-employed and unpaid family workers	1.13	1.19	1.33	0.89	1.19
**Income**	1.07	1.02	1.09	1.11 **	1.07 *
χ2	79.91	28.98	73.10	100.11	69.70
*p* value	0.000	0.004	0.000	0.000	0.000
Adjusted R^2^	0.1291	0.0152	0.1082	0.0777	0.0431

Odds of using source compared to those not using source, adjusting for all other covariates in tables. *** *p* < 0.001; ** *p* < 0.01; * *p* < 0.05.

**Table 5:** Adjusted odds ratios of correct COVID-19 knowledge by information source (*n* = 1500).

	The Main Clinical Symptoms of COVID-19 Are Fever, Coughing, and Loss of Taste.	Dinstinct from the Common Cold, Nasal Congestion, a Runny Nose, and Sneezing Are Less Common in People Infected with COVID-19.	Currently, There Is No Cure for COVID-19.	Eating or Contact with Wild Animals Would Result in Infection with the COVID-19 Virus.	Eating Kimchi Prevents COVID-19.	The COVID-19 Virus Is Airborne.	COVID-19 Is Transmitted through Respiratory Droplets.	COVID-19 Spreads from Human to Human.	Not All People with COVID-19 Will Develop Severe Symptoms.
**Number of sources**	1.02	1.09	0.95	1.03	1.16 *	1.06	0.98	1.14	1.02
**Source group**									
Traditional media	1.80 *	0.76	1.80 *	0.95	1.43	1.02	1.27	0.89	1.30
Government	1.13	1.43 *	1.01	1.04	1.02	0.89	0.86	0.66 *	0.93
Online media	2.22 **	1.09	1.96 **	1.27	0.51	1.78 *	2.65 **	2.13 **	2.17 **
Interpersonal sources	1.08	1.12	1.56 *	0.98	0.80	0.86	1.93 *	0.98	1.74 **
Doctor	0.63 *	0.72	0.68 *	1.18	1.84 **	1.07	0.64	0.64	0.68 *
χ2	206.92	42,90	86.60	85.56	60.19	74.63	283.54	261.08	182.63
*p* value	0.000	0.0102	0.000	0.000	0.0001	0.000	0.000	0.000	0.000
Pseudo R^2^	0.1344	0.0256	0.0427	0.0428	0.0589	0.0360	0.2293	0.1831	0.0982

Adjusted for all other information source variables in the model (needs, barriers), sex, age. *** *p* < 0.001; ** *p* < 0.01; * *p* < 0.05.

## Data Availability

Not applicable.
